# Superselective Embolization with Microcoil and Gelfoam for High-Flow Priapism Secondary to Bilateral Cavernous Fistulae: A Case Study

**DOI:** 10.1155/2019/3916056

**Published:** 2019-04-16

**Authors:** Sarah Prattley, Timothy Bryant, Rowland Rees

**Affiliations:** University Hospital Southampton, Tremona Road, Southampton, Hampshire SO166YD, UK

## Abstract

**Background:**

High-flow priapism is rare, and bilateral arteriocavernous fistulae formation following trauma is rarer still. Management of high-flow priapism is conservative either through observation, use of ice packs, mechanical decompression, or intracavernosal injection of *α*-adrenergic agonists, giving temporary results in selected cases. Alternatively, superselective arteriography with embolization is widely accepted. However, intervention needs to be mindful of the potential impact on long-term potency. We report the first case of bilateral arteriocavernous fistulae managed with both Gelfoam and microcoil embolization.

**Case Study:**

We present the case of a 35-year-old gentleman who attended the emergency department nine days following a fall from a moped, where he sustained bruising to his perineum, with persistent nonpainful erection. CT angiogram demonstrated bilateral arteriovenous fistulas. Management with superselective catheterisation and embolization with Gelfoam and microcoil was successful with resolution of symptoms. Long-term follow-up has shown return to normal erectile function twelve months following the injury.

**Outcomes:**

Concern regarding the effect to long-term erectile function has previously led to delayed bilateral embolization. Selection of embolization material can be tailored to the anatomical features of the fistula to help preserve function.

## 1. Introduction

High-flow priapism is rare, and bilateral arteriovenous fistulae formation following trauma is rarer still [1]. Intervention needs to be mindful of the potential impact on long-term potency [2]. We present the case of a 35-year-old gentleman who sustained bilateral arteriovenous fistulae secondary to a moped injury. Following superselective catheterisation and embolization with Gelfoam and microcoil his symptoms resolved. Long-term follow-up has shown return to normal erectile function twelve months following the injury.

## 2. Case Study

A 35-year-old gentleman presented nine days following a fall from a moped, where he sustained bruising to his perineum, but no other significant injuries. Since the incident he had developed a persistent priapism that was nonpainful, with just mild discomfort on walking. He had no lower urinary tract symptoms.

On examination there was a grade IV erection with a rigid base and shaft with slight dorsal curvature of 10 degrees, which was nontender. There was a superficial haematoma present to the scrotum and base of penis. A corporal blood gas was taken which showed pH 7.415, pCO2 5.55, and pO2 11.0, consistent with high-flow priapism.

**Figure 1:**
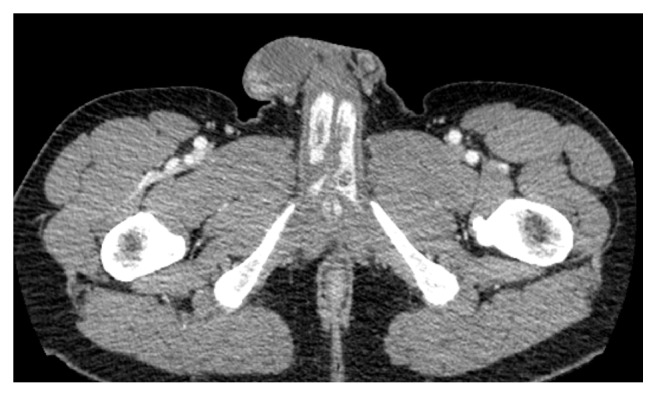
CT depicting bilateral arteriocavernous fistulae with pooling of contrast medium in the corpora.

The ultrasound Doppler did not demonstrate any evidence of fistula and had contradictory Doppler traces; therefore a CT angiogram was performed both to further evaluate the pathology and to provide anatomical arterial information. The CT demonstrated evidence of arterial pooling within the bulbar segments of the corpora cavernosa bilaterally, in-keeping with a high flow, nonischaemic priapism (Figure 1). A discussion was held with the patient regarding the options of embolization of both internal pudendal arteries versus conservative management, along with the risks of each. The patient opted for embolization.

**Figure 2:**
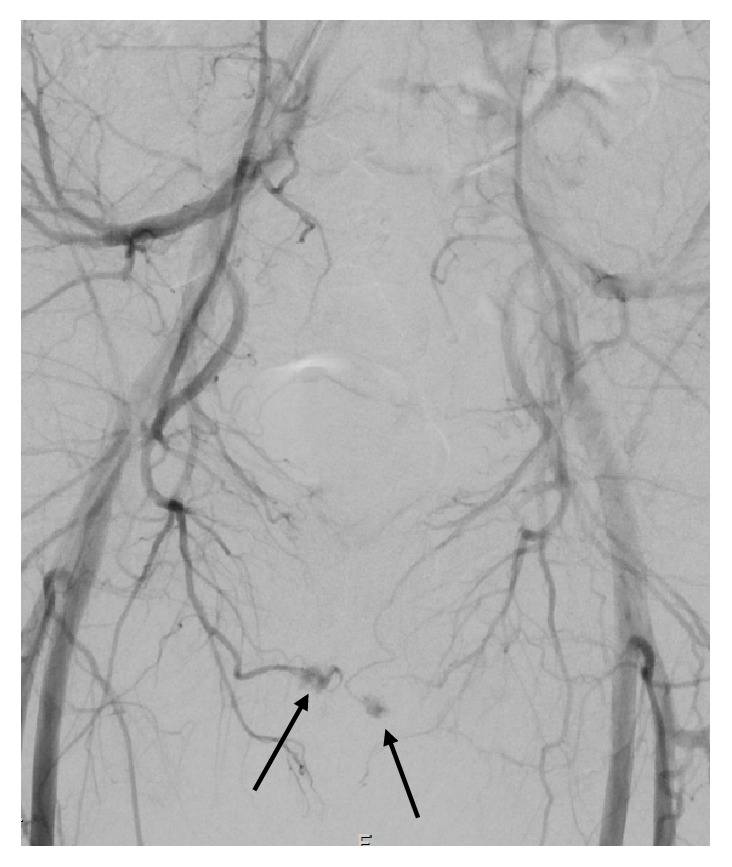
Angiography depicting extravasation of contrast at the sites of arteriocavernous fistulae.

**Figure 3:**
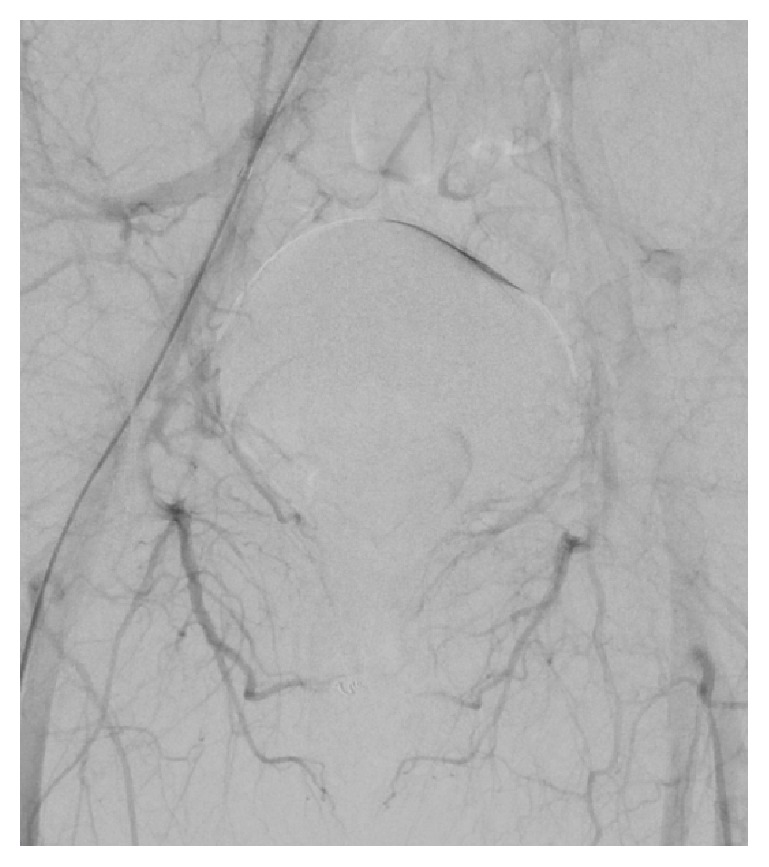
After procedure, no extravasation of contrast following successful embolization.

**Figure 4:**
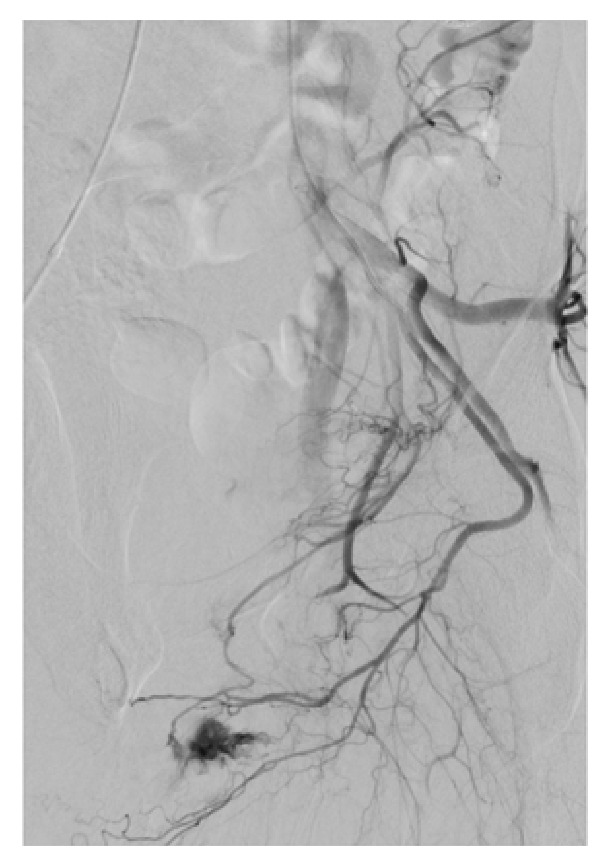
Left arteriocavernous fistula supplied by both the left internal pudendal artery and an accessory pudendal branch of the anterior prostatic artery from the obturator artery.

**Figure 5:**
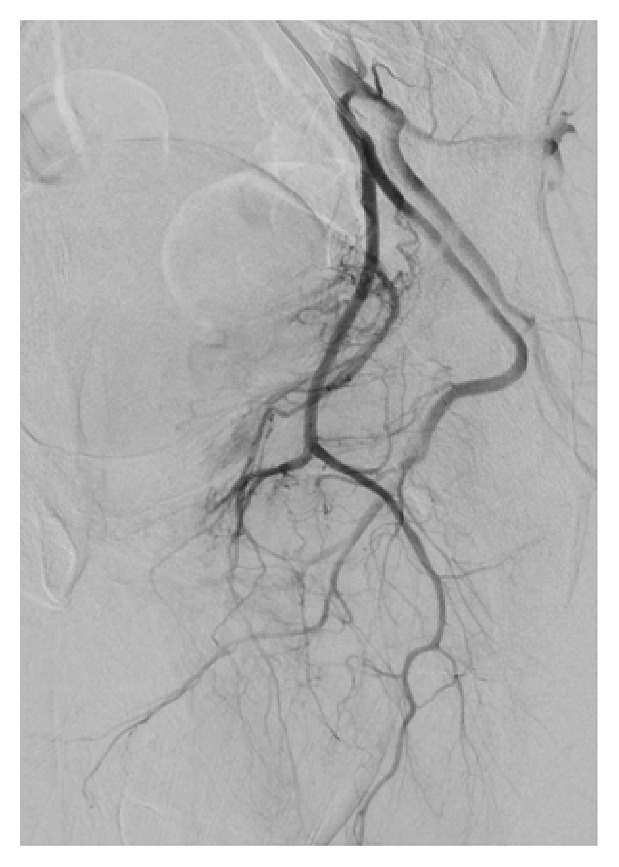
Cessation of fistula following Gelfoam embolization.

**Figure 6:**
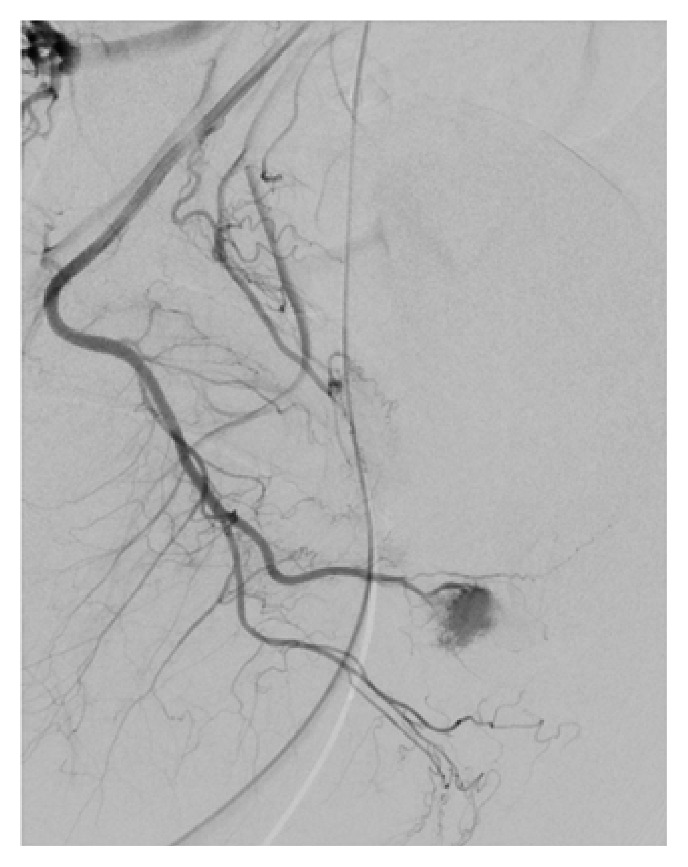
Single causative artery of arteriocavernous fistula being the distal branch of the right internal pudendal artery.

**Figure 7:**
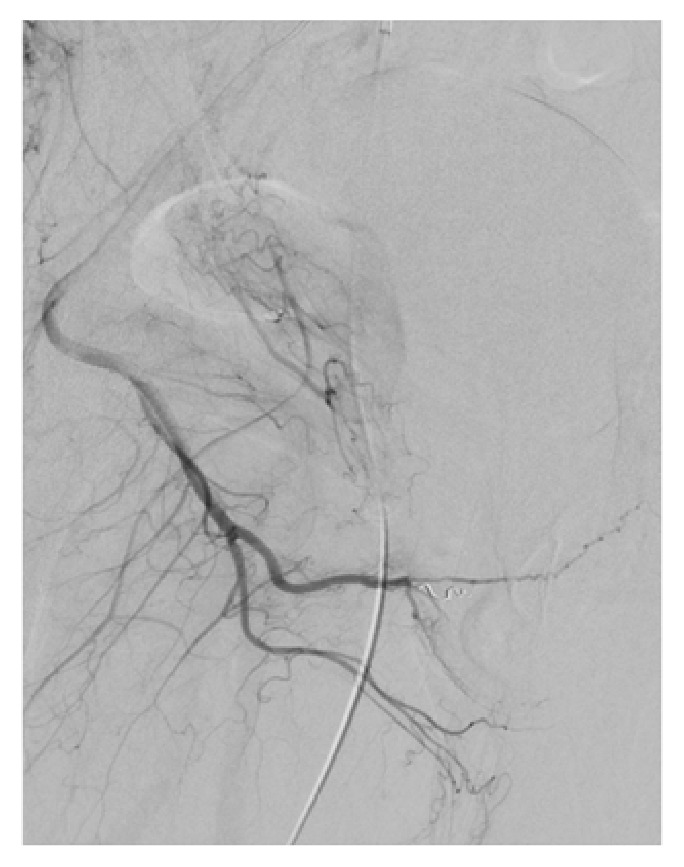
Microcoil embolization of the distal branch of the right internal pudendal artery.

The right common femoral artery was cannulated under ultrasound guidance, and aortography was performed which confirmed bilateral cavernosal fistulae. The internal pudendal arteries were bilaterally catheterised using a 4-French C2 cobra catheter. The left fistula received supply from the branches of the left internal pudendal artery and an accessory pudendal branch of the anterior prostatic artery which came off the obturator (Figures 2 and 4). Both vessels were superselectively catheterised using a 2.4fr Direxion microcatheter and 0.016 Fathom guidewire. Due to the polyarterial supply to the fistula embolization with absorbable gelatine sponge (Spongostan, Ethicon, Johnson & Johnson Medical N.V, Belgium) was selected. Absorbable gelatine sponge (Gelfoam) is temporary embolic which resorbs within four to six weeks. The sponge sheet is cut into 1-2mm pledgets which are mixed with contrast and passed through a 3-way tap to create a slurry prior to injection. This resulted in satisfactory occlusion of the fistula and persistent good antegrade flow within the nontarget vessels (Figures 3 and 5). On the right a single fistula from a distal branch of the right internal pudendal was present (Figures 2 and 6) and superselectively catheterised beyond the dorsal penile branch and embolized just proximal to the fistula using microcoils (2mm Soft platinum Type-A, Cookmedical, Bloomington, USA) (Figure 7). Postembolization angiography showed satisfactory results with no further filling of the cavernosal fistulas (Figures 3 and 7).

This patient has been sequentially reviewed in our Urology Outpatient Clinic to assess his ongoing recovery. Self-reported erectile function has slowly improved over the course of twelve months, without the need to use PDE-5 inhibitors. At one year his erections have returned to baseline.

## 3. Discussion

First described by Burt* et al. *in 1960 following traumatic coitus, high-flow priapism is typically caused by trauma resulting in damage to the cavernosal artery, or one of its branches [3]. This leads to arteriovenous fistula formation and unregulated pooling of blood within the sinusoidal space propagating a nonischaemic erection due to the continuous supply of oxygenated blood. The corpora are typically tumescent but not rigid and lack the symptom of pain caused by ischaemia and can continue for a prolonged period of time [4].

Typically, ultrasound Doppler scan demonstrated “low resistance, high-velocity” arterial waveform, with the sensitivity being nearly 100% [5]. This, however, was not the case with our patient and the colour Doppler result was not in-keeping with his clinical presentation; therefore progression to CT angiogram was necessary. The reason for this is not fully understood, but the proximal nature of the fistula may have degraded evaluation of the cavernosal arteries.

While arteriography is not used for diagnostic purposes due to its invasive nature, it is first assessed during superselective embolization for identification of the causative arteries. Penile arteriography will demonstrate outflow of the contrast medium into the corpora cavernosa from the arterial-sinusoidal fistula and will demonstrate a pooling effect (Figures 2, 4, and 6) [6]. Our patient had bilateral arteriocavernous fistulae; therefore it is essential that both sides were imaged accordingly.

Bilateral arteriocavernous fistulae formation is rare, with few cases being reported in current literature. Management of high-flow priapism is conservative either through observation, use of ice packs, mechanical decompression, or intracavernosal injection of *α*-adrenergic agonists giving temporary results in selected cases [7]. Alternatively, superselective arteriography with embolization is widely accepted [7].

There is potential concern for erectile dysfunction in the treatment of bilateral arteriocavernous fistulae particularly with the use of microcoils [2]. In order to attempt to avoid bilateral embolization, a previously reported case from Davies* et al.* found adequate detumescence with unilateral embolization despite bilateral shunting [8]. Further reported attempts at unilateral embolization have not been as successful; the studies of both Lee et al. and Gujral et al. found that unilateral embolization resulted in return of symptoms shortly following the procedure [1, 9]. Lazinger* et al. *waited 20 days between autologous blood clot embolizations of each side to see if resolution occurred; however further embolization was required [10].

Bilateral embolization has been reported with the use of both Gelfoam and microcoil with excellent results, despite the potential for erectile dysfunction. Materials such as blood autologous clots and Gelfoam are typically preferred due to the recanalization of arterial supply following temporary interruption [11]. Accurate placement can be challenging as the material is nonopaque; however, it can be mixed with contrast to aid this. The use of microcoil for bilateral embolization is more of a concern due to the potential risk of erectile dysfunction [2]; however, to date it has also yielded good results with patients returning to normal erectile function at day two and six weeks after procedure [1, 9].

The selection of embolization material for our patient was based upon the arterial supply to his fistulae. The right side was supplied by a singular cavernosal branch off the internal pudendal artery; therefore a microcoil was used. The left side, however, was supplied by at least two arteries: the distal branches of the internal pudendal and an accessory internal pudendal branch of the anterior prostatic branch which came off the obturator artery. Therefore, to allow restoration of blood flow in the long-term and to reduce the risk of erectile dysfunction Gelfoam was selected. To our knowledge this is the first reported case to use multiple materials for embolization for this presentation.

Function following bilateral embolization for all patients appears to be excellent, with restoration of normal function in all cases within a year without the need for adjuncts [1, 8–10].

Timely nature of intervention may be essential for patients with high-flow priapism in order to reduce the risk of erectile dysfunction [11]. There is currently no recommended time interval to allow conservative measures to work and for spontaneous resolution of the fistula. In a case series by Zacharakis* et al. *six patients were reviewed who underwent conservative management for 2–12 weeks following presentation, average 4.5 weeks, which eventually led to embolization as a definitive form of management. Erectile dysfunction was noted in all six patients with a reduction in IIEF-5 score, with them requiring either PDE-5 inhibitors, intracavernosal injections, or a penile prosthesis [11]. Therefore, it may be prudent that definitive intervention with superselective embolization takes place without delay to avoid potential risk of erectile dysfunction, or that the patient is aware of this risk.

## 4. Conclusion

Bilateral arteriocavernous fistula for high-flow priapism is rare; however superselective embolization with either autologous clot, Gelfoam, or microcoil appears to yield good restorative outcomes with normal levels of potency at twelve months. Potential risk of erectile dysfunction needs to be borne in mind, particularly if multiple feeding arteries are present, with selection of absorbable material being recommended in this instance. Timely intervention needs to be considered to avoid the potential risk of erectile dysfunction in the long term from conservative measures.
